# Mitochondrial Communication with Cellular Organelles in the Pathogenesis of Fatty Liver Disease in Domestic and Model Animals

**DOI:** 10.3390/ani16121800

**Published:** 2026-06-10

**Authors:** Tuoyu Geng, Amaal Omara, Ali Shoaib Moawad, Aneeqa Imtiaz, Wajeeha Tanveer, Minmeng Zhao, Jing Ge

**Affiliations:** 1 College of Animal Science and Technology, Yangzhou University, Yangzhou 225009, China; amal_emara@agr.kfs.edu.eg (A.O.); ali.shoeib@agr.kfs.edu.eg (A.S.M.); aneeqaimtiaz342@yahoo.com (A.I.); wajeehatanveer0@gmail.com (W.T.); zhaominmeng123@163.com (M.Z.); 2 Joint International Research Laboratory of Agriculture and Agri-Product Safety of the Ministry of Education of China, Yangzhou University, Yangzhou 225009, China; 3 Faculty of Agriculture, Kafrelsheikh University, Kafrelsheikh 33516, Egypt

**Keywords:** mitochondria, mitochondria-associated endoplasmic reticulum membrane, hepatic steatosis, domestic animal, organelle communication, Dairy Cow Paradox

## Abstract

Fatty liver disease represents a major metabolic disorder affecting domestic animals worldwide, with significant implications for animal health, welfare, and agricultural productivity. Disrupted communication between mitochondria and other organelles—particularly the endoplasmic reticulum, lipid droplets, and lysosomes—plays a critical role in disease pathogenesis. This review synthesizes knowledge on inter-organellar communication across domestic animals, with emphasis on species-specific adaptations. We address the “Dairy Cow Paradox”—periparturient dairy cows develop severe hepatic steatosis (>30% liver fat), yet under sterile conditions, they have a higher threshold for progressing to sterile steatohepatitis compared to rodents and humans. However, it is critical to note that severe fatty liver in dairy cows is indeed associated with impaired autophagy, inflammation, and liver damage, particularly when accompanied by ketosis or concurrent infections, and 39% of transition cows exhibit moderate to severe lymphocytic hepatitis. We propose that the tolerance to severe steatosis in dairy cows arises from three adaptations: (1) attenuated innate immune sensing via the cGAS-STING pathway; (2) enhanced lipid buffering from perilipin 5 (PLIN5) with a hypothesized ruminant-specific Val152 substitution that may stabilize lipid droplet–mitochondria contacts; and (3) dampened calcium signaling due to ER–mitochondria membrane lipid raft rigidity, elevated inositol 1,4,5-trisphosphate receptor 2 (IP_3_R2) expression, and reduced mitochondrial calcium uniporter (MCU) conductance. We contrast this with the inflammatory steatohepatitis common in rodent models driven by calcium overload and mitochondrial DNA (mtDNA) release, and glucocorticoid-mediated mitofusin 1 (MFN1) suppression, causing mitochondrial fragmentation in poultry. We identify critical knowledge gaps, including the need to define bovine and avian mitochondria-associated endoplasmic reticulum membrane (MAM) proteomes and spatially resolve hepatic zonal communication patterns. Targeting organellar communication hubs with nutraceuticals or pharmacological agents offers promising therapeutic strategies.

## Simple Summary

Fatty liver disease severely impacts the health and productivity of farm animals. This review explains how the failure of communication between mitochondria and other cell components drives this disease. We highlight key species differences, such as why dairy cows store massive liver fat without severe inflammation, the “Dairy Cow Paradox,” and why rodents and poultry are more susceptible to inflammatory injury. Understanding these protective mechanisms can guide new treatments.

## 1. Introduction

Fatty liver is arguably one of the most common and costly metabolic diseases impacting production livestock worldwide. In dairy cows, hepatic lipidosis during the periparturient period affects more than half of all high-yielding cows, and severe cases lead to lower milk production, reduced fertility, and increased culling [1,2]. Annually, the cost of this condition exceeds $60 million in the U.S. alone. Fatty liver hemorrhagic syndrome in poultry and steatosis of the liver in laying hens and broiler breeder birds also significantly affect performance and health [3]. The transition from non-alcoholic fatty liver disease to metabolic dysfunction-associated steatotic liver disease (MASLD) reflects the growing understanding of metabolic disturbances behind lipid accumulation in the liver.

The central question addressed in this review is: how does failure of communication between mitochondria and other organelles—particularly the endoplasmic reticulum (ER), lipid droplets (LDs), and lysosomes—contribute to the pathogenesis of fatty liver disease across different animal species? Mitochondria in hepatic cells provide energy sources, oxidize fatty acids, regulate calcium concentrations, and initiate apoptosis [4,5]. Contrary to their previous perception, mitochondria do not act independently and are integral parts of elaborate intracellular communication systems responsible for responding to the organism’s metabolic needs. Most such communication channels operate via membrane contact sites—regions where two membranes are attached but not merged at 10–80 nm apart [6,7]. Prominent examples include mitochondria-associated ER membranes (MAMs) that coordinate multiple biological processes (e.g., lipid metabolism, calcium exchange, and stress response) [8,9].

In high-yielding dairy cows with negative energy balance (NEB) during early lactation, rapid lipolysis leads to massive non-esterified fatty acid (NEFA) influx into the liver, which surpasses its capabilities for fatty acid oxidation and MAM function [10,11]. Consequently, multiple organellar functions become disrupted: ER stress activates the unfolded protein response (UPR), calcium homeostasis becomes disturbed, and production of reactive oxygen species (ROS) by mitochondria increases significantly [12,13]. At the same time, communication between mitochondria and lipid droplets (LDs) gets affected [14,15].

A key mystery of veterinary hepatology—which this review addresses—lies in the “Dairy Cow Paradox”: periparturient dairy cows commonly suffer from severe fatty liver disease characterized by excess accumulation of more than 30% of liver mass as triglycerides without progressing to susceptibility to sterile steatohepatitis as seen in rodent models [1,2]. By contrast, human MASLD patients with 20% liver fat typically show inflammatory infiltration and fibrosis formation. However, it is critical to note that severe fatty liver in dairy cows is indeed associated with impaired autophagy, inflammation, and liver damage when accompanied by ketosis or concurrent infections [16,17]. In fact, Pietsch et al. (2021) reported that 39% of periparturient dairy cows had moderate to severe lymphocytic hepatitis, and the degree of hepatitis was positively correlated with hepatocyte degeneration in metabotype B animals [18].

Mild fatty liver may involve enhanced autophagic activity as an adaptive response [19]. Furthermore, elevated inflammatory markers have been observed in ketotic cows prior to clinical diagnosis, suggesting that inflammation may precede and contribute to ketosis development [20,21]. We suggest that the difference in sterile inflammatory progression may be explained by (1) potential adaptations in hepatic lipid storage capacities through hypothesized PLIN5 phosphorylation regulation and increased peridroplet mitochondrial abundance [14,22,23,24]; (2) possible decreased immune recognition of mitochondrial stress in hepatocytes mediated by the cGAS-STING pathway [25,26,27]; and (3) peculiarities of MAM lipid raft composition [28,29,30].

For instance, in poultry, chronic stress arising from intensive production is associated with increased glucocorticoid production, and corticosterone induces triglyceride deposition in the liver by damaging mitochondrial fusion proteins [31]. Moreover, avian mitochondria have unique susceptibility to glucocorticoids due to glucocorticoid response elements (GREs) in the promoter region of the *MFN1* gene, not present in their mammalian counterparts [32,33], coupled with lower mitochondrial cardiolipin saturation and reduced glutathione peroxidase 1 (GPx1) activity [34,35].

This review synthesizes current knowledge on mitochondrial communication networks in the context of fatty liver disease across animal species, with particular emphasis on comparative biology spanning rodents, ruminants, poultry, and fish. We advance the hypothesis that the rate-limiting step in organellar communication failure differs by species: calcium dysregulation at MAMs predominates in murine models; very low-density lipoprotein (VLDL) export bottleneck secondary to MAM hyper-stability may limit ruminant lipid clearance; and glucocorticoid receptor-mediated *MFN1* suppression drives avian mitochondrial fragmentation.

## 2. Literature Search Methodology

To ensure transparency and reproducibility, this narrative review followed a structured literature search approach. Databases searched included PubMed, Web of Science, Scopus, and Google Scholar (February 2026; coverage 1975–2026, with emphasis on 2015–2026). Keywords combined terms related to organelle contact sites (e.g., “MAM”, “organelle contact sites”, “membrane contact sites”), fatty liver disease (e.g., “fatty liver”, “MASLD”, “NAFLD”), and species (e.g., “dairy cow”, “bovine”, “poultry”, “rodent”, “zebrafish”). Additional specific searches targeted “PLIN5/lipid droplet”, “cGAS-STING/liver”, “mitofusin/glucocorticoid”, and “IP3R/calcium signaling/hepatocyte”. Only peer-reviewed English articles (original research, reviews, clinical studies) were included; conference abstracts and book chapters were excluded. In vitro studies with mechanistic relevance to organellar communication were included. Reference lists of included articles were manually screened for additional relevant studies.

## 3. Architecture of Inter-Organellar Communication

### 3.1. Molecular Tethers

Membrane contact sites are specialized microdomains in which membranes of cellular organelles are connected via protein tethers, maintaining tight distances between the organelles’ membranes while preserving their identities [7]. The remodeling of these structures is controlled by metabolic and environmental stimuli and the cell’s energy status [36]. The composition and regulation of tethering complexes are highly conserved across species, albeit with significant differences in ruminants, rodents, and poultry.

Regarding the connection between mitochondria and ER, the main tethering complex is inositol 1,4,5-trisphosphate receptor (IP_3_R)-glucose-regulated protein 75 (GRP75)-voltage-dependent anion channel 1 (VDAC1). Here, IP_3_Rs localized on the ER interact with VDAC1 located on the surface of the mitochondrial outer membrane (OMM), mediated by the bridging chaperone GRP75 [29,37]. Species-specific differences in IP_3_R expression patterns may underlie variations in calcium dynamics. For instance, rodents mainly express IP_3_R1, while IP_3_R2/3 predominates in the bovine liver, and IP_3_R2 shows specific calcium release kinetics [28,38].

Mitofusin 2 (MFN2) is a unique protein acting as a regulator of mitochondrial fusion and ER–mitochondria tethering [39,40]. The bovine *MFN2* gene carries a ruminant-specific mutation in intron 4 that might affect alternative splicing depending on metabolic stress [39]. Another tether example is vesicle-associated membrane protein-associated protein B (VAPB)-protein tyrosine phosphatase interacting protein 51 (PTPIP51), where VAPB on the ER membrane binds PTPIP51 on mitochondria, regulating autophagy [41].

Concerning contact sites between mitochondria and LDs, PLIN5 is recognized as a key regulator of organelle interaction. Phosphorylation of PLIN5 on serine 155 regulates contact establishment. Most interestingly, the sequence around serine 155 contains a protein kinase A (PKA) docking consensus motif. In bovine PLIN5, serine 155 is preceded by valine at position 152 instead of isoleucine as in rodents. Based on sequence analysis, we hypothesize that this Val152 substitution may alter PKA docking affinity, potentially leading to more stable LD–mitochondria contacts upon lipolytic stimulation compared to rodents [14,23]. Direct functional validation of this substitution in bovine hepatocytes remains an important future goal. Additionally, oleic acid feeding results in increased PLIN5 levels and enhanced coupling of LDs and mitochondria in hepatocytes [10]. The mitoguardin 2 (MIGA2) protein forms tri-partite contacts, regulating lipid flow from mitochondrial fatty acid precursors through ER triglyceride biosynthesis to LD storage [42].

Lysosome–mitochondria contact sites are regulated by the small GTPase Rab7, which cycles between active GTP-bound and inactive GDP-bound states [43]. The mitochondrial outer membrane protein fission 1 (FIS1) recruits TBC1 domain family member 15 (TBC1D15) to promote Rab7 GTP hydrolysis, facilitating contact disassembly [44]. Peroxisome–mitochondria contacts are established through acyl-CoA binding domain containing 5 (ACBD5)-PTPIP51 interactions [45,46]. Table 1 summarizes the major tethering complexes, their functional roles, and critical species-specific variations.

### 3.2. Tools for Mapping the Hepatic Interactome

Research on organellar interaction networks has been made possible by innovative technologies. Using transmission electron microscopy (TEM), distinctive morphological changes in mitochondria, dilated ER, and increased liposomes have been identified in fatty livers of animals [47,48]. In cattle with fatty liver, TEM analysis showed mitochondrial abnormalities such as circular enlargement, fragmented cristae, and vacuolated rough ER [49].

Engineered ascorbate peroxidase (APEX) and proximity-dependent biotin identification (BioID) proximity labeling have enabled researchers to identify the MAM proteome, which includes about 1000 specific proteins in this region [8,50]. To date, however, no information is available regarding the proteome associated with bovine and avian peridroplet MAMs. Future studies should prioritize experiments with APEX2-PLIN5 proximity labeling in primary bovine hepatocytes [10,50]. Genetically encoded split fluorescent protein sensors can be used to track MAM formation and dynamics [36].

Recent advancements in spatial proteomics and transcriptomics show that roughly half of all hepatic proteins are expressed in zonation patterns [51,52]. Zonation patterns indicate that mitochondrial profiles in healthy periportal hepatocytes include increased oxidative phosphorylation (OXPHOS) with spherical mitochondria, while in pericentral regions, the number of tubular mitochondria, which synthesize lipids, increases [53]. Whether such zonation exists in bovine liver remains an open question [52,54].

## 4. Mitochondria–ER Crosstalk

### 4.1. MAMs: The Physical and Functional Interface

MAMs are highly specialized subdomains of the ER that maintain tight physical contact with the OMM at distances ranging from 10–30 nm [55,56]. MAMs serve as a platform for the coordinated action of phospholipid synthesis, calcium signaling, mitochondrial dynamics, and autophagosome biogenesis [7].

Lipid content in MAMs is distinct from the rest of the ER and the mitochondrial membrane, characterized by elevated levels of cholesterol, ceramides, and specific phospholipids [30,57]. It is hypothesized that bovine hepatic MAMs exhibit increased levels of saturated fatty acyl chains in phosphatidylethanolamine (PE) compared to rodent MAMs, potentially causing increased membrane rigidity and decreased calcium channel mobility. This might result in reduced calcium-induced stress responses in bovine liver cells despite extensive NEFA loading [10,30]. MAMs function as the primary location for the de novo biosynthesis of phosphatidylserine (PS) in hepatocytes, which is then transported to mitochondria, where it undergoes decarboxylation into PE [58].

MAM integrity is subject to dynamic regulation based on metabolic conditions. Murine models of diet-induced obesity demonstrate reduced MAM integrity due to low expression levels of MFN2 and GRP75 [28,59]. Dairy cows experiencing NEB in early lactation may generate unique MAM phenotypes where tethering protein expression remains high, but MAM phospholipid exchange activity becomes limiting for VLDL maturation—potentially representing a bottleneck for VLDL secretion that characterizes hepatic steatosis in ruminants [10].

### 4.2. Calcium Flux

Calcium transport from the ER to mitochondria is among the most important roles played by MAMs. The IP_3_R-GRP75-VDAC1 pathway acts as the primary route for this process [37,60]. Calcium is then transported to the matrix via the MCU pathway and controls dehydrogenases involved in the tricarboxylic acid (TCA) cycle [61].

Calcium oscillations are beneficial to mitochondria for energy production. However, constant calcium load results in activation of the mitochondrial permeability transition pore (mPTP), cytochrome c liberation, and initiation of apoptosis [29]. In fatty liver diseases, high lipid levels cause IP_3_Rs to become hypersensitive to normal stimulation signals, resulting in excessive calcium release [28].

The “Dairy Cow Paradox” might also be attributed to certain calcium-handling adaptations. Bovine hepatocytes have higher proportions of IP_3_R2 relative to IP_3_R1 than mouse hepatocytes. IP_3_R2 is less sensitive to IP_3_, thus increasing the requirement for IP_3_ before any calcium transport occurs—potentially helping to avoid calcium overload and mPTP opening leading to apoptosis. Moreover, based on sequence analysis, the bovine MCU possesses a single amino acid substitution (Asp261Glu) relative to the mouse sequence. If functionally confirmed, this variant might lower calcium flux conductance, potentially contributing to resistance to calcium overload in bovine hepatocytes [61,62].

Glucocorticoid-dependent downregulation of MFN1 is essential in disturbing calcium metabolism in poultry. Zhang et al. showed that continuous exposure to corticosterone causes mitochondrial fission in broiler hepatocytes, while recovery of MFN1 expression restores mitochondrial structure. In avian species, the promoter region of *MFN1* possesses a functional GRE located at −847 base pairs, which is not found in the promoter region of mammalian *MFN1* [31].

### 4.3. Phospholipid Exchange and Membrane Remodeling

MAMs are key sites for phospholipid biosynthesis and inter-organelle transport necessary for mitochondrial membrane biogenesis. PS is first synthesized in the ER, translocated to mitochondria, where it undergoes decarboxylation to PE, which is then returned to the ER to be converted to phosphatidylcholine (PC) [30,63].

Hernandez-Alvarez et al. showed that impaired ER-to-mitochondria PS delivery leads to liver disease [64]. PS is transferred from the ER to mitochondria by binding with MFN2, emphasizing the importance of MAM lipid transport processes. The PS-PE-PC shuttle may operate with slower flux rates due to MAM lipid raft stiffness in ruminants, potentially contributing to low VLDL assembly and secretion capacity [11,30,64]. The mitochondrial contact site and cristae organizing system (MICOS) complex, specifically the Mic19 subunit, has a key function in shaping cristae structure, since Mic19 depletion leads to unstructured cristae and fewer ER–mitochondria interactions [65].

### 4.4. The UPR-ROS Vicious Cycle

ER stress and mitochondrial oxidative stress share a tight interdependence mediated via MAMs. Increased protein misfolding activates three UPR receptors—inositol-requiring enzyme 1 alpha (IRE1α), protein kinase R-like ER kinase (PERK), and activating transcription factor 6 (ATF6)—all of which localize to MAMs [66]. IRE1α activation triggers increased mitochondrial Ca^2+^ uptake and ROS production [12].

Increased mitochondrial ROS generation during ER stress serves to promote UPR signaling, establishing a self-reinforcing cycle [67]. Hydrogen peroxide is produced in the ER by the oxidoreductase ERO1α during disulfide bond formation, resulting in ER- and MAM-specific local oxidative stress [68].

The UPR-induced ROS cycle in poultry may be further enhanced by mitochondrial sensitivity to redox processes. Avian mitochondria contain approximately 40% less cardiolipin compared to mammalian species, with a decreased percentage of linoleic acid incorporated into cardiolipin. Moreover, avian hepatocytes exhibit lower mitochondrial expression levels of the GPx1 antioxidant enzyme and lack the GPx4 isoenzyme dependent on selenium [34,35].

## 5. Mitochondria–Lipid Droplet Dynamics

### 5.1. Peridroplet Mitochondria

Mitochondria that form associations with LDs, known as peridroplet mitochondria (PDM), are a bioenergetically unique subset separate from cytoplasmic mitochondria (CM). This special subpopulation shows unique bioenergetic properties, proteomic profiles, and dynamics [22,69]. In the liver, PDM have increased ability to oxidize pyruvate and higher respiratory performance than CM [24].

During MASLD development, PDM abundance increases while CM decreases [24]. More than 100 protein markers are more abundant in PDM than in CM [22,50]. The expansion of PDM in dairy cattle hepatocytes may represent an adaptation to high NEFA flow. Bovine PDM proteomic analysis suggests a specific protein pattern including DGAT2, Rab18, and ACSL1 [22,24], potentially indicating specialization of bovine PDM in triglyceride production and LD formation rather than fatty acid oxidation. Docosahexaenoic acid (DHA) supplementation elevates the percentage of PDM and coupling with LDs [48].

### 5.2. Direct Fatty Acid Channeling

Mitochondria–LD contact sites enable direct transport of fatty acids from LDs to mitochondria, protecting the cytosol from lipotoxic free fatty acids [15,70].

PLIN5 controls this process—the C-terminal domain anchors on the LD surface, while the N-terminal domain attracts mitochondria [14,71]. PLIN5 acts as a docking protein for adipose triglyceride lipase (ATGL), blocking uncontrolled lipolysis and promoting selective fatty acid transfer [72]. Fatty acid transport protein 4 (FATP4) also associates with PLIN5 at contact sites to promote directed fatty acid transport [14].

The bovine PLIN5 protein harbors a single amino acid change (Val152) in the PKA consensus motif. Based on sequence analysis, we hypothesize that this may result in reduced affinity of PLIN5 for PKA compared to murine PLIN5. If confirmed, this mechanism could ensure a relatively hypophosphorylated state of PLIN5 under adrenergic stimulation, thereby maintaining LD–mitochondria contacts even during lipolytic signaling [14,23,72]. This hypothesized molecular adaptation might allow cows to meet the metabolic challenges of milk production—namely, partitioning large quantities of fatty acids between oxidation and re-esterification.

### 5.3. DRP1-Mediated Fission

Mitochondrial fission tends to occur preferentially at contact sites with the ER [73]. Fission also occurs at LD sites when lipids are in excess. DRP1 associates with LD contact sites and induces fission of both LDs and mitochondria [74].

**Figure 1:**
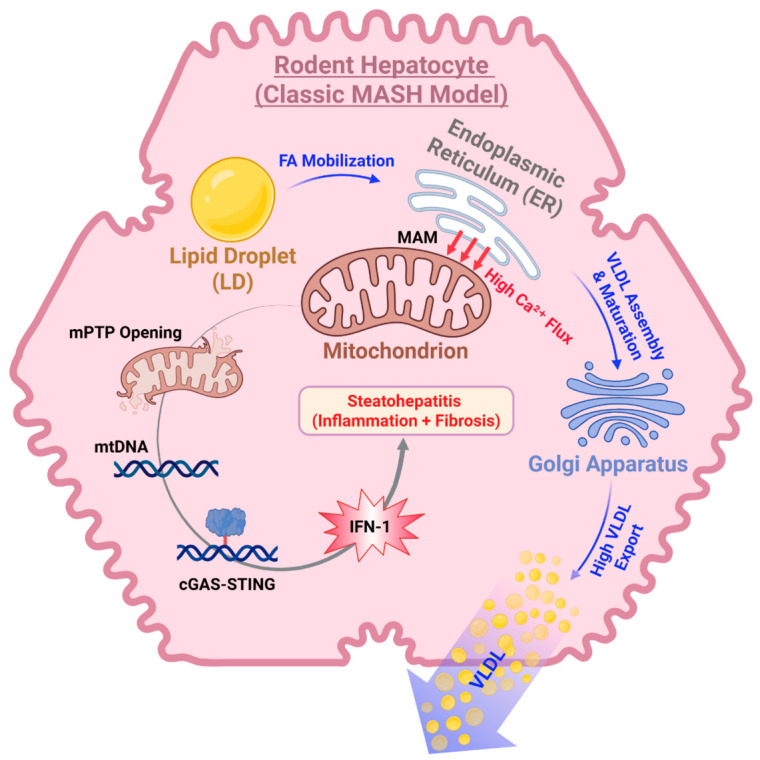
Steatohepatitis in Rodents (The Inflammatory Model). In rodent hepatocytes, steatosis and inflammation are coupled. Fatty acids are directed to the ER for VLDL assembly and secretion (thick blue arrows), maintaining a moderate LD burden. At MAMs, pathological Ca^2+^ flux through the IP_3_R-GRP75-VDAC1 complex triggers mPTP opening and mtDNA release. Cytosolic mtDNA activates cGAS-STING signaling, driving type I interferon production and progressive steatohepatitis with fibrosis. Abbreviations: MAM, mitochondria-associated ER membrane; mPTP, mitochondrial permeability transition pore; mtDNA, mitochondrial DNA; cGAS, cyclic GMP-AMP synthase; STING, stimulator of interferon genes; VLDL, very low-density lipoprotein. Created in BioRender. Ge, J. (2026) https://BioRender.com/yl9iplx (accessed on 7 June 2026).

As MASLD progresses, DRP1 and FIS1 are upregulated, while MFN2 and optic atrophy 1 (OPA1) are downregulated, leading to mitochondrial fragmentation and dysfunction [75]. Figure 1 illustrates the canonical rodent model where lipotoxicity induces calcium overload in MAMs, leading to mtDNA release and inflammation.

Mitochondrial fission in poultry is highly regulated by glucocorticoids. The avian *DRP1* gene promoter has two active GRE sites that induce transcriptional activation upon corticosterone exposure. In contrast, the avian *MFN1* gene promoter has one active GRE site that leads to transcriptional inhibition [31]. As a result, mitochondrial networks become fragmented within 6–12 h of stress. Mammals lack functional GRE sites for *DRP1* and *MFN1/2* genes.

### 5.4. Comparative Anatomy: Ruminant, Rodent, and Avian Species

Rodent Hepatocytes: Rodents possess many small LDs (0.5–2 μm), efficient VLDL packaging machinery, and highly motile MAMs. In mouse MASLD models, MAM structural integrity is disrupted early, marked by decreased MFN2 and GRP75 levels [28]. Rodent hepatocytes elicit vigorous inflammatory responses to mitochondrial damage through cGAS-STING and NLRP3 pathways [27].

Ruminant Hepatocytes (Bovine): Bovine hepatocytes are characterized by a small number of large LDs (>5 μm), representing an adaptation to continuous hepatic lipid transport [1,11]. Ruminants have inherent restrictions in hepatic triglyceride output as VLDL due to: (1) diminished microsomal triglyceride transfer protein (MTTP) activity and gene expression; (2) low apolipoprotein B (APOB) production rate; and (3) potentially MAM lipid raft stiffness hindering phospholipid exchange for VLDL maturation. The inability to export VLDL results in increased likelihood of steatosis, but under sterile conditions, it may slow progression to steatohepatitis [1,11].

**Figure 2:**
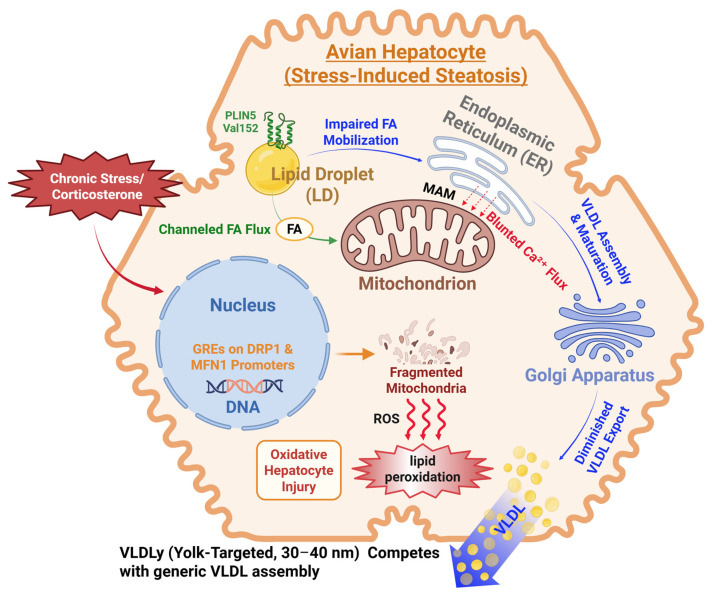
Oxidative Steatosis in Poultry (The Stress-Driven Model). In avian hepatocytes, chronic stress drives steatosis toward oxidative injury through glucocorticoid-mediated mitochondrial fragmentation. Corticosterone-activated glucocorticoid receptor binds GREs on *DRP1* and *MFN1* promoters (avian-specific, absent in mammals), simultaneously inducing fission and suppressing fusion to fragment mitochondria within 6–12 h. Fragmented mitochondria are intrinsically vulnerable due to approximately 40% lower cardiolipin content and reduced GPx1 expression, leading to excessive reactive oxygen species (ROS) production and oxidative hepatocyte injury. VLDL export is further impaired by competition from yolk-targeted VLDLy particles. These combined defects predispose poultry to fatty liver hemorrhagic syndrome. Abbreviations: MAM, mitochondria-associated ER membrane; DRP1, dynamin-related protein 1; GRE, glucocorticoid response element; MFN1, mitofusin 1; ROS, reactive oxygen species; FA, fatty acids; VLDL, very low-density lipoprotein; VLDLy, yolk-targeted very low-density lipoprotein. Created in BioRender. Ge, J. (2026) https://BioRender.com/bj23dao (accessed on 7 June 2026).

Avian Hepatocytes: Avian hepatocytes show specific organellar architecture, including a special VLDL type called “yolk-targeted” VLDL (VLDLy), which is smaller (30–40 nm) than regular VLDL and contains apolipoprotein VLDL-II, making it resistant to lipoprotein lipase. This VLDL secretion pathway may compete with general VLDL formation under metabolic stress, resulting in excess hepatic lipid storage [76,77]. Broilers have higher susceptibility to hepatic steatosis under chronic stress. Avian hepatocyte mitochondria have unique vulnerability to cortisol-driven fragmentation due to GREs in *MFN1* and *DRP1* promoters, plus intrinsically low cardiolipin and GPx antioxidant capacities. Additionally, birds have intrinsically low cardiolipin and GPx antioxidative capacities (Figure 2). Table 2 summarizes comparative features of hepatic mitochondrial populations.

### 5.5. The Dairy Cow Paradox: Mechanisms of Relative Protection from Progression to Sterile Steatohepatitis

Periparturient dairy cows provide a unique natural model of extreme hepatic steatosis. During the first 4–6 weeks of lactation, high-producing dairy cows mobilize up to 60 kg of adipose tissue, releasing massive quantities of NEFA. The liver takes up NEFA in proportion to blood concentration, leading to triglyceride accumulation exceeding 30% of liver wet weight in severe cases [1,2]. Despite this profound steatosis, cows do not progress to susceptibility to sterile steatohepatitis as rodent models do under experimental conditions. However, it is critical to note that severe fatty liver is associated with impaired autophagy, inflammation, and liver damage [16,17]. Pietsch et al. (2021) documented that 39% of transition cows had moderate to severe lymphocytic hepatitis, with inflammatory changes accentuated in cows exhibiting poor silage quality (metabotype B) [18]. Furthermore, concurrent bacterial infections (mastitis, metritis) can trigger steatohepatitis. The ‘paradox’ refers specifically to the reduced incidence and slower progression of sterile inflammatory responses compared to rodents, not the complete absence of inflammation.

We propose the following three hypothesized mechanisms as potential explanations for why severe hepatic steatosis in periparturient dairy cows may not invariably progress to sterile steatohepatitis as susceptibly as in rodents:

Mechanism 1 (hypothesized): Attenuation of cGAS-STING signaling. Mitochondrial damage leading to mtDNA liberation and subsequent cGAS activation, STING-dependent signaling, and Type I interferon production has been established in mice and humans with MASLD [25,27]. Based on preliminary evidence, basal STING expression in bovine liver may be lower than in rodents, and the promoter region of the bovine *STING* gene contains a ruminant-specific sequence insertion with a putative inhibitory function [25,27]. Furthermore, sequence alignment reveals an Arginine to Histidine substitution in bovine cGAS at a residue critical for DNA binding, leading to the hypothesis that bovine cGAS may have intrinsically reduced affinity for mtDNA [26]. Direct quantitative validation of these activities in primary bovine cells is needed.

Mechanism 2 (hypothesized): PLIN5-mediated enhancement of lipid buffering. Based on sequence analysis, the bovine PLIN5 Val152 substitution may reduce phosphorylation by PKA, thus potentially preserving LD–mitochondria interactions and channeled fatty acid transport under adrenergic stimulation [14,23,72]. Increased PDM abundance in bovine steatosis may further compartmentalize fatty acid oxidation within discrete mitochondrial subregions, potentially decreasing ROS formation and mtDNA damage. This could represent a “containment” response where extensive hepatic lipid accumulation occurs without consequential organelle dysfunction [10,22,24,25,78].

Mechanism 3 (hypothesized): Stiffness and calcium retention by bovine MAMs. Due to their distinct lipid content, bovine MAMs may have decreased membrane fluidity, potentially inhibiting calcium channel mobility. Combined with the high IP_3_R2:IP_3_R1 ratio and the predicted MCU Asp261Glu mutation, bovine hepatocytes may have high intrinsic capacity to withstand mitochondrial calcium overload and prevent mPTP opening [28,29,30,38]. As mPTP opening is critical for mtDNA release and NLRP3 inflammasome activation, such calcium buffering could prevent sterile inflammation [27,79].

While these hepatocyte-autonomous adaptations provide a compelling hypothetical framework, systemic factors may also contribute to the “Dairy Cow Paradox.” The unique ruminant metabolic profile—including reliance on propionate for gluconeogenesis and a distinct circulating NEFA composition during NEB—may further modulate hepatic inflammatory responses. A less lipotoxic profile of circulating fatty acids could directly influence MAM fluidity independently of the protein adaptations described. The interplay between systemic and cell-intrinsic factors remains an important area for future investigation.

**Figure 3:**
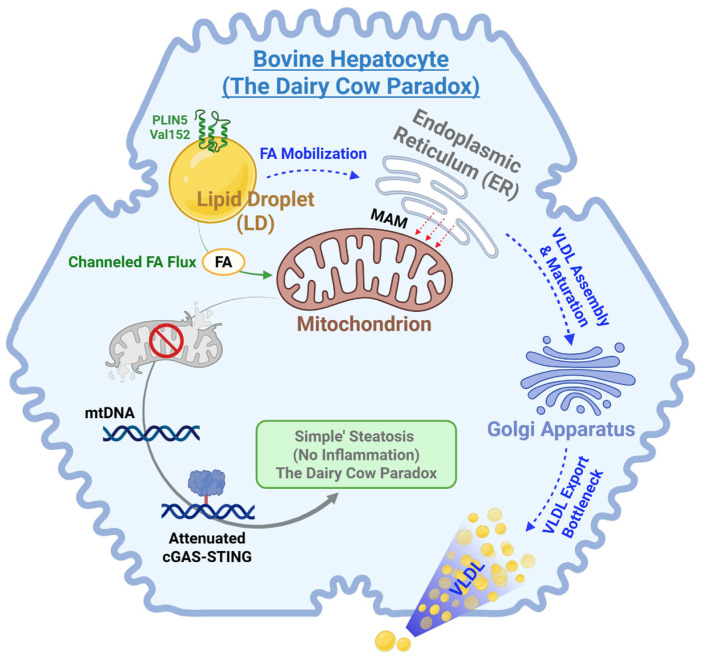
Simple Steatosis Without Inflammation (The Dairy Cow Paradox). In periparturient bovine hepatocytes, massive steatosis exceeding 30% liver fat develops without inflammatory progression due to three protective adaptations. First, intrinsically low MTTP/APOB capacity and rigid MAM phospholipids create a VLDL export bottleneck (blue dotted arrow), promoting lipid storage. Second, a PLIN5 Val152 substitution stabilizes LD-mitochondria contacts and channels fatty acids into peridroplet mitochondria, limiting lipotoxic free fatty acid release. Third, attenuated cGAS-STING signaling—via low STING expression, a cGAS Arg231His mutation reducing mtDNA binding, and blunted MAM Ca^2+^ flux (elevated IP_3_R2:IP_3_R1 ratio, MCU Asp261Glu substitution)—collectively prevents mitochondrial damage-induced inflammation. However, inflammation (including lymphocytic hepatitis in 39% of cows) does occur, particularly with concurrent infection or poor feed quality (metabotype B). Abbreviations: MAM, mitochondria-associated ER membrane; PLIN5, perilipin 5; FA, fatty acids; VLDL, very low-density lipoprotein. Created in BioRender. Ge, J. (2026) https://BioRender.com/7roeyoc (accessed on 7 June 2026).

In summary, these protective adaptations may help explain why periparturient steatosis in dairy cows does not invariably advance to sterile steatohepatitis at the same incidence as in rodents. However, concurrent bacterial infections can circumvent this higher inflammatory threshold, resulting in steatohepatitis when fatty liver is severe [1,2]. Furthermore, as demonstrated by Pietsch et al. (2021), poor silage quality leading to more severe negative energy balance (metabotype B) is associated with accentuated inflammatory, degenerative, fibrotic, and proliferative changes in the liver [18]. Figure 3 illustrates the hypothesized protective adaptations.

### 5.6. Inflammation in Ketotic and Fatty Liver Cows

The relationship between hepatic lipid accumulation and inflammation in dairy cows is context-dependent. In cows with mild fatty liver (triglyceride content 1–5%), enhanced autophagic activity may serve as an adaptive mechanism to maintain cellular homeostasis and prevent progression to more severe disease [19]. These cows show increased formation and degradation of autophagosomes, which may help clear damaged organelles and limit inflammatory signaling. In contrast, cows with severe fatty liver (>10% triglycerides) exhibit impaired autophagic activity, characterized by accumulation of p62, LC3-II, and ubiquitinated proteins, along with increased autophagosome numbers—findings indicative of blocked autophagic flux [16]. This impairment is associated with elevated circulating inflammatory markers, including haptoglobin, serum amyloid A, and lipopolysaccharide-binding protein, as well as increased liver enzyme activities (AST, ALT, GLDH, GGT).

Pietsch et al. (2021) comprehensively evaluated histomorphologic changes in 80 German Holstein cows throughout the transition period and reported that 39% of cows had moderate to severe lymphocytic hepatitis [18]. The degree of fatty infiltration was positively correlated with the severity of liver cell degeneration, and moderate to severe lymphocytic infiltration of the liver was observed across all sampling time points. Importantly, cows with greater lactation numbers (≥5) had perisinusoidal fibrosis more often than younger cows. The authors identified three metabotypes based on alterations in the liver metabolome between antepartum and postpartum status. Metabotype B animals—which calved during a period when grass silage quality was presumably decreased—exhibited a higher chance of fatty infiltration, lower glycogen storage, perisinusoidal fibrosis, and positive correlations between increased fat deposition and marked glycogen depletion, as well as increased degenerative, inflammatory, fibrotic, and proliferative changes of hepatic tissue [18].

Ketotic cows demonstrate further evidence of hepatic inflammation and cell death. Elevated serum concentrations of TNF-α, IL-6, and IL-1β have been reported, along with increased hepatocyte apoptosis mediated by the MAPK-p53-Nrf2 pathway [80]. More recently, necroptosis—a programmed form of necrotic cell death—has been identified in the livers of ketotic cows, with activation of the RIPK1/RIPK3/MLKL signaling axis [17]. TNF-α has been identified as an inducer of necroptosis in bovine hepatocytes, potentially driving cellular injury and inflammatory responses.

Importantly, elevated circulating markers of inflammation (LPS, LBP, SAA, haptoglobin) have been observed in ketotic cows prior to clinical diagnosis, suggesting that inflammation may precede and potentially contribute to the development of ketosis rather than being merely a consequence [20,21]. This has led to the ‘leaky gut’ hypothesis, whereby increased intestinal permeability during the periparturient period may allow translocation of LPS into circulation, triggering systemic inflammation and metabolic dysregulation. Bobe et al. (2004) similarly emphasized that fatty liver is associated with impaired immune function, including decreased lymphocyte proliferation, reduced neutrophil phagocytosis, and increased susceptibility to infectious diseases such as mastitis and metritis [1].

In summary, the available evidence—including the foundational review by Bobe et al. (2004) and the comprehensive histopathological study by Pietsch et al. (2021)—demonstrates that inflammation is indeed associated with hepatic lipidosis in dairy cows [1,18]. The “Dairy Cow Paradox” should therefore be understood as a relative protection from progression to sterile steatohepatitis compared to rodents, not as a complete absence of inflammation.

## 6. Mitochondria–Lysosome Interplay

### 6.1. Mitophagy and Lipophagy

Mitochondria–lysosome contacts modulate mitochondrial dynamics, Ca^2+^ fluxes, and organelle turnover [43,44]. Contact formation and disassociation are controlled by Rab7 GTPase; active Rab7-GTP increases contact stability, while TBC1D15 (recruited by FIS1 from the mitochondrial membrane) stimulates conversion to inactive Rab7-GDP and contact disassociation [44].

Mitophagy impairment is characteristic of MASLD; both the PINK1/Parkin pathway and BNIP3/NIX receptor pathway are downregulated [81,82,83]. Mitophagic activity may be preserved or even increased in bovine hepatocytes during early-stage steatosis. This could relate to the “Dairy Cow Paradox” and potentially prevent release of pro-inflammatory mtDNA. Unlike mice with metabolic dysfunction-associated steatohepatitis (MASH), Rab7 expression may be stable in MASLD cattle [81,82,83].

Lipophagy is diminished in MASLD. Low Rab7 expression inhibits LD delivery to lysosomes, while decreased lysosome-associated membrane protein 2A (LAMP2A) levels limit chaperone-mediated autophagy of LD coat proteins [84,85].

### 6.2. Lysosomal Iron and Fe-S Cluster Biogenesis

Mitochondria–lysosome contact sites regulate iron metabolism and Fe-S cluster assembly [43,86]. The mucolipin TRPML1 protein in lysosomes is responsible for divalent iron ion efflux, which is directly supplied to mitochondria via contact sites.

MASLD-associated lysosomal acidification dysfunction impairs TRPML1 activity and iron efflux, leading to mitochondrial iron insufficiency [87,88]. Chondroitin sulfate correction of lysosomal acidification dysfunction restores iron delivery to mitochondria [87]. Ferredoxin reductase expression is increased in humans and mice with MASLD [89].

### 6.3. AMPK-mTORC1 Nutrient Sensing

The AMPK-mTORC1 signaling axis acts as a master regulator of organellar communication, integrating nutrient status to coordinate autophagy, mitochondrial biogenesis, and lysosomal function across multiple organelles. The antagonism of AMPK and mTORC1 is crucial to the central hub of nutrient sensing, where organellar biogenesis and autophagy are coordinated [90,91].

Dysregulation of this nutrient-sensing hub disrupts inter-organellar communication in MASLD. The balance of this pathway is altered in MASLD with paradoxical AMPK inactivation and mTORC1 overactivation in conditions of energy abundance [92]. The transition period in dairy cows is a phase of severe energy deficiency, which should induce AMPK signaling. However, the presence of elevated insulin and other anabolic hormones can pose contradictory signals. Wang et al. showed that disturbances in the hindgut microbial balance contribute to postpartum energy metabolism abnormalities by blocking acetate-induced AMPK-peroxisome proliferator-activated receptor alpha (PPARα) hepatic signaling [93]. Since AMPK directly regulates mitophagy and mitochondrial quality control, its inactivation further impairs organelle communication networks. Restoration of AMPK activity, such as via DHA supplementation [48], offers a promising solution to improve mitochondrial function in transition cows.

## 7. Auxiliary Axes: Peroxisome and Nuclear Communication

### 7.1. Peroxisomal–Mitochondrial Cooperation

Peroxisomes and mitochondria cooperate in fatty acid β-oxidation, each with distinct substrate specificity. Peroxisomes have higher affinity for very long-chain fatty acids (VLCFAs); their oxidation produces shortened chains that can undergo further oxidation in mitochondria [94].

ACBD5 acts as an interorganelle bridge between mitochondria and peroxisomes via PTPIP51 and VAPB proteins [45,95]. Peroxisome–mitochondria contact increases under oxidative stress, allowing efficient ROS transfer between organelles [46]. Peroxisomal β-oxidation is compromised in MASLD, with decreased acyl-CoA oxidase 1 (ACOX1) [96]. In poultry, heat stress negatively affects peroxisome function; heat-stressed broilers exhibit increased ACBD5 protein levels, potentially reflecting compensatory upregulation of peroxisome–mitochondria contacts [45,46].

### 7.2. UPRmt and Epigenetic Remodeling

The mitochondrial unfolded protein response (UPRmt) represents a critical communication axis between mitochondria and the nucleus, allowing mitochondrial stress to trigger adaptive transcriptional responses that influence organellar function. UPRmt constitutes a retrograde signaling pathway conveying signals about mitochondrial stress to the nucleus. Translocation of transcription factors such as activating transcription factor 5 (ATF5), C/EBP homologous protein (CHOP), and CCAAT/enhancer-binding protein beta (C/EBPβ) to the nucleus activates the UPRmt pathway. Activation of UPRmt results in increased expression of mitochondrial chaperones and proteases [97].

Epigenetic remodeling at the mitochondria–nucleus interface further modulates this communication. The UPRmt process entails regulation of gene expression by way of an epigenetic mechanism. Two demethylases, namely jumonji domain-containing protein 3 (JMJD3) and PHD finger protein 8 (PHF8), are stimulated to enhance chromatin remodeling of the UPRmt target genes. UPRmt controls sirtuin 3 (SIRT3), a histone deacetylase enzyme that uses nicotinamide adenine dinucleotide (NAD+) as its substrate to catalyze the deacylation process [98]. Activation of UPRmt in MASLD occurs in two phases involving compensatory induction, followed by downregulation [13].

Mitochondrial metabolites themselves serve as signaling molecules that communicate metabolic status to the nucleus via epigenetic mechanisms. Metabolites produced by the mitochondria have been shown to regulate gene expression in the nucleus via epigenetic means. Acetyl-CoA, a product of mitochondrial beta-oxidation, functions as an activator for histone acetylation [99]. Decreased activity of beta-oxidation of fatty acids in MASLD leads to insufficient amounts of acetyl-CoA, which interferes with histone acetylation. Thus, impaired mitochondrial fatty acid oxidation directly compromises nuclear gene expression programs. Mitochondrial DNA, in turn, has been epigenetically modified in MASH patients; the gene encoding NADH dehydrogenase subunit 6 (*ND6*) shows methylation [100].

## 8. Pathological Consequences of Dysregulated Communication

### 8.1. Calcium Overload and mPTP Induction

Mitochondrial calcium overload due to MAM dysfunction leads to mPTP opening—a non-specific channel spanning both mitochondrial membranes that, upon activation, dissipates mitochondrial membrane potential (MMP), halts ATP production, and releases pro-apoptotic factors such as cytochrome c [101].

In MASLD, IP_3_R calcium release upregulation favors mPTP activation and cell death [28]. MAM enrichment in high-fat diet (HFD)-fed animals is positively associated with calcium overload and liver cell damage [102]. Cyclophilin D (CypD) acts as an essential mPTP regulator and forms part of the IP_3_R-GRP75-VDAC protein complex within MAMs [79].

As discussed in Section 5.5, bovine cells may inherently resist calcium overload owing to hypothesized rigid lipid rafts in MAMs, increased IP_3_R2:IP_3_R1 ratios, and the predicted MCU Asp261Glu mutation. These potential calcium-buffering mechanisms could inhibit mPTP opening and cell death. This is consistent with reports that despite severe fatty liver in periparturient dairy cattle, hepatocyte apoptosis remains relatively low compared to mouse MASH models [49].

### 8.2. mtDNA Release and the cGAS-STING Pathway

mtDNA released into the cytosol from damaged mitochondria is a potent damage-associated molecular pattern (DAMP) triggering the cGAS-STING pathway. In MASLD patients, mitophagy impairment results in damaged mitochondrial accumulation and mtDNA release, activating cGAS-STING [25,27].

STING activation in Kupffer cells and hepatic stellate cells promotes pro-inflammatory cytokine and chemokine production, driving hepatic inflammation and fibrosis progression [27]. STING knockdown or pharmacological inhibition significantly decreases inflammation, steatosis, and fibrosis in mouse MASH models [103].

Attenuation of cGAS-STING signaling in bovine hepatocytes (Section 5.5), if confirmed, could represent an evolutionary adaptation decoupling steatosis from sterile steatohepatitis.

### 8.3. Cellular Senescence

Cellular senescence represents the pathological endpoint of sustained organellar communication failure, where persistent mitochondrial-lysosomal crosstalk dysfunction creates a self-perpetuating cycle of damage. Senescence occurs in the liver during the progression of MASLD and promotes fibrosis and hepatocellular carcinoma (HCC). The major triggers for senescence include mitochondrial dysfunction and defective mitophagy [104,105].

The failure of organelle communication between mitochondria and lysosomes creates a destructive feedback loop. The failure of mitochondrial and lysosome removal leads to a feedback loop where damaged mitochondria become persistent sources of ROS, which damage lysosomes [87,88]. This impaired inter-organellar crosstalk prevents clearance of dysfunctional mitochondria, exacerbating oxidative stress and cellular damage. Hepatic senescence is associated with a senescence-associated secretory phenotype (SASP), which involves the release of inflammatory cytokines and matrix metalloproteinases responsible for activating hepatic stellate cells [106]. Notably, SASP factors can propagate organellar dysfunction to neighboring cells, amplifying the pathological communication failure across the liver. The elimination of senescent cells by senolytic drugs ameliorates steatosis, inflammation, and fibrosis in rodent models [107,108]. These therapeutic successes confirm that interrupting the organelle communication failure at the level of senescent cell clearance can reverse disease progression. Table 3 illustrates the detrimental effects of abnormal organelle interactions.

## 9. Insights from Animal Models and Comparative Biology

### 9.1. Murine MASH Models

Murine models have significantly advanced understanding of MAM defects in MASLD development. A study of 39 murine MASLD models showed that Western diet models were comparable to human MASH, while choline-deficient models inducing rapid MASH-fibrosis were not metabolically relevant [109]. In murine MASH models, MAM dysfunction includes lower MFN2 and GRP75 levels [59,64].

Arruda et al. found that increased ER–mitochondria contact enrichment in the liver led to MAM-related insulin resistance and mitochondrial dysfunction in obese mice [28]. Exogenous MAM components caused MAM induction, while IP_3_R2 activity inhibition improved glucose homeostasis and prevented hepatic steatosis. Talari et al. provided comprehensive data on CM and PDM content changes during MASH development, showing a negative correlation between CM and PDM [24].

However, despite their value for exploring MASLD molecular mechanisms, murine models have limited translation to veterinary practice due to significant MAM structure and function differences [11,109].

### 9.2. The Periparturient Dairy Cow

The periparturient dairy cow is a unique model of intensive lipolysis and hepatic steatosis, mobilizing up to 60 kg of fatty acids from adipose tissue within 4–6 weeks. Elevated circulating NEFAs may be 5–10-fold higher, with one-third of NEFAs taken up by the liver [1].

The hypothesized molecular mechanisms of the “Dairy Cow Paradox” (Section 5.5) include: (1) possible downregulation of the cGAS-STING pathway; (2) increased PLIN5 expression for fat storage via the Val152 residue and PDM expansion; and (3) enhanced MAM rigidity and calcium homeostasis through IP_3_R2 dominance and predicted MCU Asp261Glu mutation. Du et al. showed that periparturient dairy cows with mild steatosis possess more mitochondria as a compensatory strategy [49]. Wang et al. found upregulated DGAT2 in periparturient cows with fatty liver, indicating greater triglyceride synthesis ability [110].

Zhang et al. found that rumen-protected DHA supplementation (180 g/d) reduced liver lipid accumulation, improved liver function, reduced oxidative stress and inflammation, and increased insulin sensitivity in periparturient dairy cows [48]. TEM indicated that DHA improved mitochondrial ultrastructure and increased mitochondria–LD interactions.

### 9.3. Zebrafish Imaging

*Danio rerio* zebrafish serve as a model organism for organelle physiology studies due to optical clarity and high manipulability. Transgenic zebrafish lines with fluorescent protein reporters for specific organelles enable investigation of organelle formation, dynamics, and interactions in living organisms [111].

Overfeeding zebrafish larvae leads to MASLD development within 10 days, with lipids accumulating primarily in pericentral regions [111]. CRISPR/Cas9 knockout of obesity genes shows that mitochondrial dynamics and LD metabolism regulation are relevant to MASLD in zebrafish. *Pink1* knockout zebrafish exhibit ceramide accumulation, increased mitophagy, and β-oxidation defects [112], resembling mammalian conditions.

## 10. Nutritional Drivers of Organellar Dysfunction

### 10.1. Dietary Patterns and Hepatic Steatosis

The quantity and composition of dietary intake directly shape organellar communication networks in the liver. High-energy diets exceeding energy requirements promote hepatic steatosis through multiple mechanisms: (1) increased de novo lipogenesis via SREBP-1c activation in the ER; (2) suppression of fatty acid oxidation through PPARα downregulation; and (3) impaired MAM integrity due to excess lipid accumulation [28,59]. In dairy cows, overfeeding during the dry period is a well-established risk factor for periparturient fatty liver, with cows fed > 1.65 Mcal NE_1_/kg DM showing higher liver triglyceride content postpartum [1,2].

Dietary carbohydrate composition profoundly influences hepatic organelle function. High-starch diets (e.g., excessive grain feeding) increase ruminal production of propionate, which is converted to glucose in the liver via gluconeogenesis. This process generates excess acetyl-CoA that can be diverted to triglyceride synthesis when oxidative capacity is exceeded [11]. In poultry, high-energy, low-protein diets induce fatty liver hemorrhagic syndrome by upregulating de novo lipogenesis in the ER while simultaneously impairing mitochondrial VLDL assembly [76,77].

### 10.2. Macronutrient-Specific Effects on Organelle Communication

Carbohydrates: Excessive carbohydrate intake, particularly fructose and sucrose, directly stresses ER-mitochondria communication. Fructose metabolism in hepatocytes rapidly depletes ATP, generating uric acid and ROS that impair MAM calcium transfer [12]. High-glucose conditions upregulate IP_3_R expression, sensitizing MAM calcium release and promoting mPTP opening under lipotoxic stress [28].

Lipids: The composition of dietary fatty acids dictates their effects on organelle communication. Saturated fatty acids (SFAs) such as palmitate are particularly lipotoxic, incorporating into MAM phospholipids and increasing membrane rigidity. This impairs the PS-PE-PC shuttle required for VLDL assembly, contributing to the ruminant VLDL export bottleneck [10,30]. Unsaturated fatty acids (UFAs) such as oleate promote LD-mitochondria tethering by upregulating PLIN5 expression, enhancing fatty acid channeling into oxidative pathways [10,14]. Omega-3 polyunsaturated fatty acids (DHA, EPA) are incorporated into MAM phospholipids, increasing membrane fluidity and normalizing PS-PE-PC cycling, which explains the therapeutic effects of DHA supplementation in transition dairy cows [48].

Protein: Dietary protein restriction impairs VLDL synthesis by limiting apolipoprotein B (APOB) availability. In dairy cows, inadequate metabolizable protein during the transition period exacerbates fatty liver because APOB synthesis cannot keep pace with triglyceride export demands [11]. Certain amino acids have direct signaling functions: leucine activates mTORC1 at the lysosomal surface, while arginine and glutamine modulate AMPK activity and autophagy [90,91].

### 10.3. Micronutrient Regulation of Organelle Communication

Vitamins: Vitamin A (retinol) is essential for MAM integrity, as retinol-binding protein (RBP4) is synthesized at ER-mitochondria contacts. Vitamin E (α-tocopherol) protects MAM phospholipids from peroxidation, preserving calcium transfer capacity [30]. Vitamin D regulates VDAC1 expression, modulating mitochondrial calcium uptake [29].

Minerals: Calcium is the central signaling ion at MAMs; hypocalcemia in periparturient cows directly impairs IP_3_R-GRP75-VDAC1 complex function [29,79]. Magnesium is required for ATP-dependent calcium pump activity in the ER. Selenium is critical for GPx1 and GPx4 expression, which protect mitochondria from oxidative damage; selenium deficiency in poultry exacerbates fatty liver hemorrhagic syndrome [34,35]. Iron must be properly trafficked via mitochondria-lysosome contacts; iron dysregulation impairs Fe-S cluster biogenesis and mitochondrial respiration [43,86].

### 10.4. Nutritional Interventions That Modulate Organelle Communication

Dietary supplementation strategies that target organelle communication have shown promise. Rumen-protected DHA (180 g/d) in transition dairy cows increases MAM fluidity, enhances LD-mitochondria tethering, and upregulates TOMM20 and MtCo-1 expression, indicating improved mitochondrial biogenesis [48]. Choline supplementation supports PC synthesis at MAMs, facilitating VLDL export. Betaine (trimethylglycine) restores methionine metabolism and SAMe-dependent methylation reactions involved in MAM phospholipid synthesis [30]. For a detailed discussion of nutraceuticals (silymarin, berberine, curcumin, coenzyme Q10), see Section 13.2.

### 10.5. Species-Specific Nutritional Considerations

Ruminants: The rumen microbiome modifies dietary nutrients before hepatic delivery. High-grain feeding increases ruminal LPS production, which translocates to portal blood and activates Kupffer cell TLR4 signaling, inducing inflammation that secondarily impairs MAM function [20,21]. Propionate from starch fermentation is gluconeogenic but also activates PPARγ in adipose tissue, promoting adiposity [11].

Poultry: Lipogenic capacity is high, and birds are susceptible to diet-induced steatosis. High-energy, low-protein diets induce fatty liver hemorrhagic syndrome by upregulating SREBP-1 and downregulating PPARα, disrupting MAM lipid exchange [76,77].

Rodents: High-fat, high-sucrose diets are standard models for MASLD. These diets upregulate CD36-mediated fatty acid uptake, overwhelm mitochondrial β-oxidation, and promote ER stress with compensatory UPR activation that eventually fails [12,28].

## 11. Systemic Integration of Organelle Communication

While this review focuses on intracellular organelle communication, it is essential to acknowledge the broader systemic context. The liver does not function in isolation; its metabolic state is profoundly influenced by endocrine signals, adipose tissue crosstalk, and gut-derived factors.

Endocrine Regulation: Insulin, glucagon, glucocorticoids, and growth hormone all modulate hepatic mitochondrial function. During the periparturient period in dairy cows, insulin resistance in adipose tissue promotes lipolysis, while the liver remains insulin-sensitive, creating a metabolic mismatch that drives NEFA uptake and steatosis [1,2].

Adipose Tissue–Liver Axis: Adipose tissue lipolysis releases NEFA and glycerol, with NEFA serving as both an energy source and a signaling molecule. Adipokines such as leptin and adiponectin directly influence hepatic mitochondrial function and inflammation [78].

Gut–Liver Axis: The ‘leaky gut’ hypothesis proposes that increased intestinal permeability during the periparturient period allows LPS translocation into portal circulation, activating Kupffer cells and promoting hepatic inflammation [20,21]. Bile acids, gut microbiota metabolites, and dietary components further modulate liver function through enterohepatic circulation. A comprehensive understanding of fatty liver pathogenesis requires integration of these systemic factors with organelle-level mechanisms.

## 12. Diagnostic Approaches to Fatty Liver Disease in Veterinary Practice

### 12.1. Biochemical Markers

Traditional serum biomarkers for hepatic steatosis include liver enzymes (AST, ALT, GLDH, GGT), though these reflect hepatocellular damage rather than lipid accumulation per se. In dairy cows, elevated NEFA (>0.4 mM) and BHB (>1.2 mM) are associated with increased risk of fatty liver and ketosis, though these reflect systemic energy balance rather than hepatic lipid content directly [1,2].

Acute phase proteins (haptoglobin, serum amyloid A, lipopolysaccharide-binding protein) are elevated in cows with severe fatty liver and ketosis, serving as markers of inflammation-associated metabolic dysfunction [20,21].

### 12.2. Imaging Modalities

Ultrasonography has emerged as a valuable non-invasive tool for assessing hepatic lipid content. Liver echogenicity increases with fat accumulation due to increased acoustic impedance at lipid-water interfaces. Studies have demonstrated a correlation between ultrasound image texture analysis and liver total lipid content in dairy cows [21], enabling on-farm diagnosis using standard ultrasound equipment.

### 12.3. Liver Biopsy and Histopathology

Liver biopsy remains the gold standard for definitive diagnosis, allowing direct triglyceride quantification (normal <1%, mild 1–5%, moderate 5–10%, severe >10% wet weight) and histopathological assessment of inflammation, fibrosis, and hepatocellular changes. However, biopsy is invasive and carries risks of hemorrhage and infection [1].

### 12.4. Emerging Biomarkers

Recent advances have identified novel biomarkers associated with mitochondrial dysfunction and organelle stress, including circulating mtDNA fragments as DAMPs [113,114], fibroblast growth factor 21 (FGF21) [115,116], and growth differentiation factor 15 (GDF15) [117]. In dairy cows, impaired mitophagy markers (PINK1, Parkin) correlate with ROS overproduction in fatty liver [118]. These hold promise for non-invasive assessment of mitochondrial health in production animals.

### 12.5. Species-Specific Considerations

Diagnostic approaches must be adapted to each species. In dairy cows, the transition period (3 weeks prepartum to 3 weeks postpartum) is the critical window for monitoring. In poultry, fatty liver hemorrhagic syndrome is typically diagnosed post-mortem, though ultrasound and biochemical screening are being developed [3]. Rodent models allow for terminal histology and molecular analyses not feasible in production animals [109].

## 13. Therapeutic Targeting of Organelle Communication

### 13.1. Pharmacological Modulation

Pharmacological interference with MAM tethers is emerging as a novel therapy. Urolithin A enhances calcium regulation via sarco/endoplasmic reticulum Ca^2+^-ATPase (SERCA) function at MAMs [119]. Statins lower PLIN5 levels by interfering with sterol regulatory element-binding protein 2 (SREBP2), altering mitochondrial-LD connectivity. While this enhances lipolysis, the net effect on steatohepatitis is context-dependent and requires careful evaluation [120]. The antisense oligonucleotide ION224 targeting DGAT2 has shown marked improvements in liver steatosis and histopathology in MASH patients in Phase II trials [121].

Metformin increases VDAC1 expression in MAMs, maintaining mitochondrial Ca^2+^ regulation and limiting ROS production [122]. In chickens, MFN1 expression restoration can restore mitochondrial architecture and inhibit lipid deposition, suggesting mitochondrial fusion-promoting measures may help prevent stress-induced fatty liver [31]. Glucocorticoid receptor antagonists also merit consideration.

### 13.2. Nutraceuticals and Phytomedicine

Nutraceutical interventions offer attractive strategies for managing mitochondrial dysfunction. Silymarin activates the Nrf2 pathway, improving antioxidant status; studies show significant decreases in ALT, AST, and hepatic steatosis [123,124].

Berberine indirectly activates AMPK via mitochondrial complex I inhibition, promoting fatty acid oxidation and mitochondrial biogenesis [125]. Coenzyme Q10 ameliorates liver steatosis and endothelial dysfunction in MASLD [126]. Curcumin induces AMPK signaling while inhibiting NF-κB-mediated inflammation and oxidative stress; nanoformulations show enhanced bioavailability [127].

Omega-3 polyunsaturated fatty acids (DHA, EPA) inhibit SREBP-1 and activate PPARα, decreasing de novo lipogenesis and increasing fatty acid oxidation [128]. In dairy cattle, rumen-protected DHA supplementation decreases liver lipid deposition, improves mitochondrial function, and increases TOMM20 and MtCo-1 transcription [48]. This effect may be explained by DHA incorporation into MAM phospholipids, increasing membrane fluidity and normalizing PS-PE-PC cycling for VLDL assembly [30,48].

Recent advances in phytomedicine targeting MASLD include polyphenol-rich extracts (resveratrol, quercetin, epigallocatechin gallate), which modulate mitochondrial function and reduce oxidative stress. Microbiome-based interventions—probiotics, prebiotics, and fecal microbiota transplantation—are gaining attention for their ability to reduce gut-derived endotoxemia and systemic inflammation.

### 13.3. Exercise and Caloric Restriction

Lifestyle modifications benefit organellar network remodeling. Moderate-intensity aerobic activity increases MFN2 levels at PDM, improves LD–mitochondria interaction, and enhances fatty acid metabolism in mouse MASLD models [129]. The same regimen restores normal UPRmt regulation and increases FGF21 release [13].

Caloric restriction decreases ceramide concentrations and boosts mitochondrial synthesis via SIRT3-mediated SOD2 activation [130,131]. For dairy cattle, management practices optimizing dry matter intake during the transition period can alleviate NEB and fatty liver development. DHA supplementation [48] and dietary interventions favoring hindgut acetate formation to activate AMPK-PPARα signaling [93] partially mimic positive outcomes associated with mitochondrial physiology. For chickens, environmental enrichment and stress minimization through corticosterone reduction may prevent GRE-mediated MFN1 repression and mitochondrial fission.

## 14. Future Directions and Knowledge Gaps

### 14.1. Knowledge Gaps

Several key questions remain unanswered. First, the MAM proteome in bovine and avian species is unknown; priority should be given to APEX2-PLIN5 proximity labeling in primary bovine hepatocytes exposed to oleate to elucidate ruminant-specific PDM interaction networks [10,50]. Second, whether zonation in protein distribution observed in rodents exists in bovine liver requires investigation; spatial transcriptomics of liver biopsy samples from transition cows could reveal zone-dependent MAM tethering protein expression [51,52,54]. Third, the role of mtDNA release and cGAS-STING activation in poultry fatty liver disease requires study; CRISPR/Cas9 generation of *STING* knockout chickens could help determine corticosterone effects on lipid storage [27,31,103]. Fourth, the role of non-parenchymal cells in hepatic organelle communication remains underexplored in veterinary species [43,44].

### 14.2. The Next Frontier: Spatial Omics and 3D Imaging

Combining spatial transcriptomics, proteomics, and metabolomics with advanced imaging will enable a breakthrough understanding of organellar communication. Key experiments for veterinary species include: (1) focused ion beam scanning electron microscopy (FIB-SEM) of transition cow hepatocytes to determine PDM-LD contact site morphology [24,49]; (2) cryo-electron microscopy (cryo-EM) tomography of avian mitochondria under normal and corticosterone-treated conditions [31]; and (3) spatial lipidomics of bovine liver lobules using MALDI-MSI focusing on MAM-specific phospholipids [30,57].

## 15. Limitations

The authors acknowledge several important limitations of this review.

### 15.1. Reliance on Indirect and Inferential Evidence

Several mechanistic hypotheses presented—particularly the functional consequences of the bovine PLIN5 Val152 substitution, the MCU Asp261Glu mutation, and the proposed attenuation of cGAS-STING signaling—are based primarily on sequence analysis and indirect evidence from heterologous systems. Direct functional validation in primary bovine hepatocytes or in vivo models has not yet been performed. Readers are cautioned that these hypotheses require experimental confirmation.

### 15.2. Challenges in Interspecies Extrapolation

While comparative analysis offers valuable insights, extrapolation between species is complicated by fundamental differences in metabolism, liver architecture, and regulatory mechanisms. Findings from rodent models—the most extensively studied system—may not translate directly to ruminants or poultry, and vice versa.

### 15.3. Limited In Vivo Validation

Many organelle communication mechanisms discussed have been characterized primarily in cell culture systems. The extent to which these processes operate identically in intact animals, particularly under physiological conditions such as periparturient NEB, remains to be determined.

### 15.4. Potential Bias of Narrative Review

As a narrative review rather than a systematic review or meta-analysis, this work is subject to potential selection bias in the inclusion and interpretation of literature. The authors have endeavored to present a balanced perspective but acknowledge that alternative interpretations of the available data exist.

## 16. Conclusions

Based on the presented findings, we propose that fatty liver disease in veterinary animals results from impairment of organellar communication networks rather than malfunction of individual organelles. Mitochondria act as key regulators of information flow originating from ER, LDs, lysosomes, and peroxisomes at specific membrane contacts [7,36]. Importantly, the proposed rate-limiting factor for organellar communication malfunction may vary by species. For rodents, disruption of calcium balance in MAMs leads to IP_3_R-GRP75-VDAC1 pathway overactivity, mPTP opening, mtDNA leakage, and cGAS-STING-mediated inflammation [25,27,28]. For ruminants, impaired VLDL export resulting from hypothesized MAM hyper-stability and inherent APOB/MTTP expression restrictions may cause massive steatosis; however, the same MAM hyper-stability and potential calcium adaptation mechanisms may provide relative protection against sterile steatohepatitis—offering a potential explanation for the “Dairy Cow Paradox” [1,11,30,49]. For poultry, glucocorticoid-mediated transcriptional control of mitochondrial dynamics via GRE activation causes MFN1 downregulation and DRP1 upregulation, which, combined with low GPx antioxidant and cardiolipin content, triggers steatosis [31,34,35].

The “Dairy Cow Paradox” illustrates how inflammation may be relatively attenuated despite pronounced fatty changes under sterile conditions. However, it is critical to emphasize that severe fatty liver in dairy cows is indeed associated with impaired autophagy, inflammation, and liver damage when accompanied by ketosis or concurrent infections, and histopathologic studies document moderate to severe lymphocytic hepatitis in up to 39% of transition cows. The ability to understand and potentially recapitulate protective measures in species that lack them represents an exciting opportunity for future research. We emphasize the need to characterize MAM proteomes across species, apply spatial omics to production animal tissues, and develop genetically manipulable avian models.

## Figures and Tables

**Table 1:** Major Inter-Organellar Tethering Complexes in Hepatocytes with Species-Specific Divergence.

Contact Site	Tethering Complex	Primary Function	Species-Specific Divergence	References
ER-Mitochondria	IP_3_R-GRP75-VDAC1	Calcium transfer	Bovine: Elevated IP_3_R2:IP_3_R1 ratio; Avian: Uncharacterized	[29,31,37]
ER-Mitochondria	MFN2-MFN1/2	Structural tethering, fusion	Bovine: Ruminant-specific intron 4 insertion; Avian: *MFN1* promoter contains GRE	[31,39,40]
ER-Mitochondria	VAPB-PTPIP51	Autophagy regulation	Conserved across mammals; avian uncharacterized	[41]
Mitochondria-LD	PLIN5	Fatty acid channeling	Bovine: Val152 substitution (hypothesized to alter PKA docking affinity)	[14,23]
ER-Mito-LD	MIGA2-VAPA/B	Three-way lipid coordination	Ruminant/Avian: unexplored	[42]
Mitochondria-Lysosome	Rab7-TBC1D15	Contact dynamics, fission	Bovine: Rab7 expression preserved in steatosis	[43,44]
Peroxisome-Mitochondria	ACBD5-PTPIP51	VLCFA transfer, ROS signaling	Avian: ACBD5 elevated under heat stress	[45,46]

**Table 2:** Comparative Features of Hepatic Mitochondrial Subpopulations Across Species.

Feature	Rodent PDM	Bovine Hepatocytes	Avian Hepatocytes	References
Size/Morphology	Larger, elongated	Enlarged in steatosis; PDM expand	Fragmented under stress; reduced cristae density	[22,31,49]
Bioenergetics	High pyruvate oxidation	Compensatory increase then decline	Decreased MMP with corticosterone	[24,31,49]
Key Proteins	MFN2, PLIN5 (Ile152)	DGAT2, PLIN5 (Val152)	MFN1 suppressed by GR; DRP1 induced	[10,14,31]
Disease Response	PDM increase then decrease	Mitochondrial number increases then declines	Rapid fragmentation; ROS-driven injury	[10,24,31,49]
MAM Composition	Fluid, dynamic	Hypothesized: rigid lipid rafts; high saturation	Low cardiolipin; GPx-deficient	[28,30,31]
Calcium Handling	IP_3_R1-dominant; sensitive	IP_3_R2-dominant; hypothesized buffered; MCU Asp261Glu	GRE-driven dysregulation	[28,29,31]
Inflammatory Propensity	High (STING-active)	Low-moderate (STING-suppressed?)	Moderate (stress-amplified)	[25,27,31]

**Table 3:** Pathological Consequences of Dysregulated Organelle Communication in Fatty Liver Disease.

Pathological Event	Molecular Mechanism	Cellular Consequence	Species-Specific Features	References
Calcium overload	Enhanced IP_3_R-GRP75-VDAC1 Ca^2+^ transfer	mPTP opening, loss of ΔΨm, cytochrome c release	Bovine: hypothesized buffered by IP_3_R2/MCU adaptations; Avian: GRE-driven dysregulation	[28,29,31]
mtDNA release	Mitochondrial damage, impaired mitophagy	cGAS-STING activation, type I IFN production	Bovine: hypothesized STING-suppressed; Avian: uncharacterized	[25,27]
ROS overproduction	ERO1α-IP_3_R1 oxidation, ETC dysfunction	Oxidative stress, lipid peroxidation	Avian: amplified by low cardiolipin/GPx; Bovine: hypothesized buffered by PDM sequestration	[31,67,68]
Failed mitophagy	PINK1-Parkin pathway inhibition	Accumulation of damaged mitochondria	Bovine: Rab7 preserved; Avian: GR-driven impairment	[44,81,82,83]
Cellular senescence	MAM expansion, DNA damage	Cell cycle arrest, SASP	Bovine: low incidence in steatosis alone; Avian: uncharacterized	[105,106]

## Data Availability

Data sharing is not applicable to this article as no datasets were generated or analyzed in the current study.

## Abbreviations

The following abbreviations are used in this manuscript:

MAM	Mitochondria-Associated Endoplasmic Reticulum Membrane
MASLD	Metabolic Dysfunction-Associated Steatotic Liver Disease
MASH	Metabolic Dysfunction-Associated Steatohepatitis
ER	Endoplasmic Reticulum
LD	Lipid Droplet
PDM	Peridroplet Mitochondria
ROS	Reactive Oxygen Species
UPR	Unfolded Protein Response
PLIN5	Perilipin 5
IP_3_R	Inositol 1,4,5-Trisphosphate Receptor
GRP75	Glucose-Regulated Protein 75
VDAC1	Voltage-Dependent Anion Channel 1
MFN1/2	Mitofusin 1/2
MCU	Mitochondrial Calcium Uniporter
mPTP	Mitochondrial Permeability Transition Pore
mtDNA	Mitochondrial Deoxyribonucleic Acid
cGAS	Cyclic GMP-AMP Synthase
STING	Stimulator of Interferon Genes
DRP1	Dynamin-Related Protein 1
VLDL	Very Low-Density Lipoprotein
NEFA	Non-Esterified Fatty Acid
NEB	Negative Energy Balance
GRE	Glucocorticoid Response Element
GPx	Glutathione Peroxidase
DGAT2	Diacylglycerol O-Acyltransferase 2
AMPK	AMP-Activated Protein Kinase
PPARα	Peroxisome Proliferator-Activated Receptor Alpha
